# A 3-D Surface Reconstruction with Shadow Processing for Optical Tactile Sensors

**DOI:** 10.3390/s18092785

**Published:** 2018-08-24

**Authors:** Hanjun Jiang, Yan Yan, Xiyang Zhu, Chun Zhang

**Affiliations:** Institute of Microelectronics, Tsinghua University, Beijing 100084, China; thuyyin@126.com (Y.Y.); zhuxy15@mails.tsinghua.edu.cn (X.Z.); zhangchun@tsinghua.edu.cn (C.Z.)

**Keywords:** tactile sensor, surface reconstruction, 3-D reconstruction, shadow detection, robotic finger

## Abstract

An optical tactile sensor technique with 3-dimension (3-D) surface reconstruction is proposed for robotic fingers. The hardware of the tactile sensor consists of a surface deformation sensing layer, an image sensor and four individually controlled flashing light emitting diodes (LEDs). The image sensor records the deformation images when the robotic finger touches an object. For each object, four deformation images are taken with the LEDs providing different illumination directions. Before the 3-D reconstruction, the look-up tables are built to map the intensity distribution to the image gradient data. The possible image shadow will be detected and amended. Then the 3-D depth distribution of the object surface can be reconstructed from the 2-D gradient obtained using the look-up tables. The architecture of the tactile sensor and the proposed signal processing flow have been presented in details. A prototype tactile sensor has been built. Both the simulation and experimental results have validated the effectiveness of the proposed 3-D surface reconstruction method for the optical tactile sensors. The proposed 3-D surface reconstruction method has the unique feature of image shadow detection and compensation, which differentiates itself from those in the literature.

## 1. Introduction

Tactile sensing is required in the touch-enabled applications, such as surgical manipulators, artificial limb prostheses, and humanoid robots, to understand the objects’ interaction behavior [1,2]. The key design challenges of the tactile sensing technique include the miniaturization, system integration, spatial resolution, sensitivity, response speed, robustness, etc. [2,3,4]. There has been significant advancement in the tactile sensor technologies in the past decade [3,4], including the sensors based on the pressure sensitive elements [5], the capacitive force sensing arrays [6], the pressure to image sensing elements [7], and the piezoelectric oxide semiconductor field effect transistors [8]. The optical tactile sensors have the advantage of high resolution, such as the GelSight sensor in [9] with a resolution of 320 × 240. The tactile surface property perception for the object recognition is one of the significant tasks of the tactile sensors [3,4]. This work focuses on the tactile surface shape reconstruction which can be further used for the surface texture recognition and object type identification.

On the basis of sensor technologies, there has also been remarkable progress in the tactile property perception and recognition methods, among which the machine learning based methods are the most popular. The principal component analysis method was used in [10] for the paper object recognition using a micro-cantilevers based multiaxial tactile sensor. The machine learning methods including the naive Bayes algorithm, the decision tree, and the naive Bayes tree were used to distinguish different materials based on their surface texture in [11]. A recurrent spiking neural network using a semi-supervised approach was proposed by the authors of [12] for the tactile surface texture classification. For active touch sensing, the touch exploration strategy is extremely important. The Bayesian exploration strategy was utilized in [13] to improve the tactile object recognition performance. Recently, Kaboli M., et al., have made significant progress in developing the active touch algorithm which selects the most informative exploratory action for the multi-modal robotic skin, and their work can significantly improve the data efficiency for a certain learning accuracy [14,15]. The active tactile transfer learning algorithm in [16,17] exploited its prior tactile knowledge to learn about new unknown objects with a further reduced number of data samples. The set of tactile descriptors proposed in [18] has been proven effective for various learning based object recognition methods with the tactile object exploration.

As stated above, this work focuses on the tactile surface 3-D reconstruction. There are some reported methods for a robotic finger to perceive the surface shape/texture information in the literature. The BioTac method [19,20] used the incompressible liquid to convey the deformation from the object surface to a pressure transducer, which could sense the vibrations as small as a few nanometers. The tactile fingertip (TACTIP) method [21,22,23,24] was a biologically inspired sensing method, based on the deformation of the epidermal layers of the human skin. As a contrast, the optical methods based on the image processing have the advantages of directly providing intuitive information about the object surface properties and the high resolution [25,26,27,28]. For example, A. Maldonado and H. Alvarez presented an optical tactile sensor for the object surface texture recognition in [25], in which the texture information was extracted from the gray-scale images using the Gray Level Co-Occurrence Matrices. Nevertheless, the optical tactile sensors in literature usually ignore the image shadow which exists for the object surface with large fluctuation and may degrade the 3-D reconstruction performance. This work focuses on the tactile surface reconstruction based on the optical tactile sensing, and the shadow problem is particularly studied.

In this paper, an optical tactile sensor is reported which is capable of reconstructing the 3-D surface of the objects manipulated by the robotic fingers. Similar to the GelSight sensors [9,29,30,31,32,33], the tactile sensor in this work mainly consists of an elastic sensing layer to transform the object surface texture into the deformation information, and an image sensor together with multiple flashing lights with different illumination directions to record the deformation information. This work attempts to solve the shadow issue associated with the point light sources used in this design, especially for the situation where there exists the large and steep fluctuation on the object surface. With the independently controlled flashing LED lights and the proposed image processing method, the tactile sensor in this work recognizes the image shadow first, and then handles the points not in the shadow and those in the shadow in two different ways. An algorithm based on the depth from deformation gradient has been implemented to reconstruct the 3-D surface information from the images taken which include the shadow information.

The paper is organized as follows. The hardware implementation of the tactile sensor is presented in Section 2. The image processing flow for the 3-D surface reconstruction is given in Section 3. The implementation results are provided in Section 4. Finally, this work is concluded in Section 5.

## 2. Tactile Sensor Hardware Architecture

**Figure 1:**
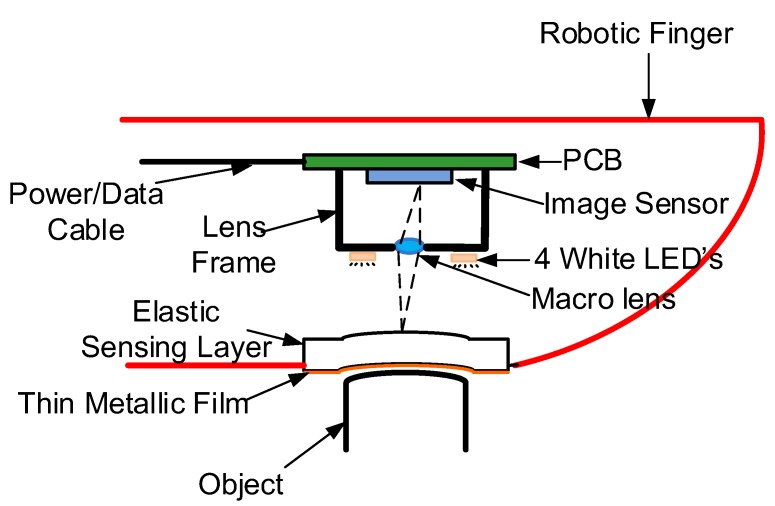
Optical tactile sensor architecture (conceptual).

The conceptual architecture of the tactile sensor for the application of robotic fingers is shown in Figure 1. It mainly consists of an elastic sensing layer made of the soft silicone, an image sensor and 4 white LEDs evenly spaced surrounding the image sensor lens for the illumination. Compared with the previous work reported in [34], there are no markers required on the elastic sensing layer, which are actually not easy to manufacture. The outside of the sensing layer is covered with a thin metal film. The thin film will exhibit deformation when the robotic finger touches an object, and the deformation is correlated to the object surface shape. The images of the film deformation are taken from the inner side of the sensing layer which is transparent. The sensing film is illuminated by the LEDs that project from different directions, and 4 images of the same film deformation are captured by switching on the 4 LEDs one by one. The 3-D surface of the object surface can then be reconstructed from the 2-D film deformation images.

To implement the prototype sensor, experiments have been conducted to find the optimal ratio of the silicone and the curing agent in the sensing layer. The final choice is to have the silicone to curing agent ratio to be 30:1. An aluminum or copper thin film with the thickness of 3000 angstroms is deposited on the outside of the silicone sensing layer using the sputtering method. The thickness has also been decided through experiments for the compromise between the deformation image quality and the thickness uniformity. For any object, the tactile sensor takes deformation images of the sensing layer, instead of the direct images of the object. Since the sensing film has evenly distributed bidirectional reflectance distribution function (BRDF) independent of the objects, the surface reconstruction is no longer affected by the optical properties of the object surface.

In this work, the 3-D shape of the object surface area which is pressed on the tactile sensor is reconstructed, which is similar to the GelSight sensors. There are several typical methods for the 3-D reconstruction from the 2-D images. This work adopts the photometric stereo method proposed by Woodham [35] which was also used in the GelSight sensors. The photometric method uses the reflectance maps in the form of look-up tables (LUT’s) to estimate the surface local gradients. The reflectance maps correlate the observed image intensity with the surface geometry gradients. It is wanted that for each point on the object surface, the measured image intensity is immune to any disturbance.

**Figure 2:**
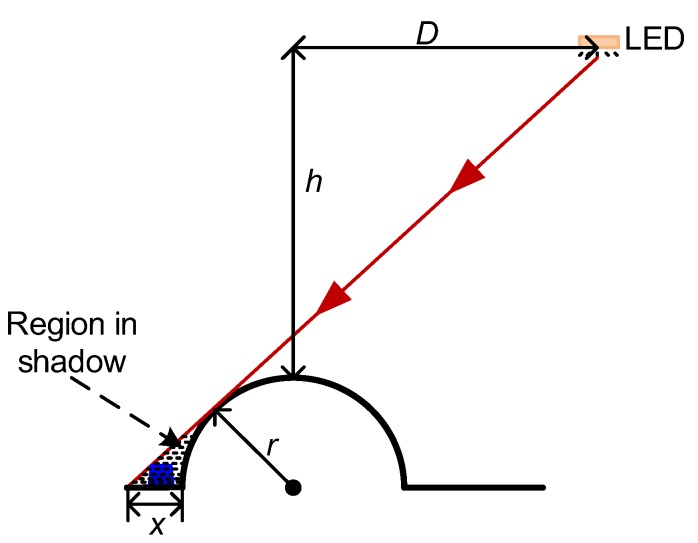
Shadow caused by a point light source.

The early versions of GelSight sensors used several discrete surface mounted LEDs as the illumination lights [30]. The recently reported GelSight sensors tried to improve the illumination uniformity by replacing each LED with a LED array [32] which could be viewed as a surface light source. However, due to the limited space inside the small robotic finger in this design, only 4 discrete LEDs are used, which can only be viewed as the point light sources. A point light source illuminating from a fixed direction may cause the shadow, especially for an object with large and steep fluctuation on the surface. As shown in Figure 2, for the spherical pump with a radius of *r* on the object surface, if a point light source with the height of *h* and the horizontal distance of *D* is used, there exists a shadow region with the length of *x*, which is approximately given by:

(1) $$x\approx r\left(\sqrt{1+\frac{D^{2}}{h^{2}}}-1\right)$$

For the tactile sensor shown in Figure 1, the maximum value of *D* is close to *h*. If *D* = *h* = 5 mm and *r* = 1 mm, we have *x* ≈ 0.4 mm, which is relatively large for a sensing area of about 20 mm. If any small shape, for instance, the blue shape in Figure 2, falls in the shadow region, the measured image intensity is obviously affected by the shadow. The image shadow turns to be the disturbance for this specific shape, and the image with this light source cannot be used to reconstruct the height of this specific shape. Note that four LEDs project from four different directions. For any point on the object surface, usually at most one of the LEDs may cause the shadow, and the height information of that point can still be reconstructed using the images illuminated by the other three LEDs.

Following the strategy above, for each object to identify, the proposed tactile sensor takes four images by switching on the four LEDs one by one in order to provide the illumination from four different directions. The unique procedure of this work is to check whether any point (pixel) is in the shadow in one of the four images before the inverse reflectance mapping. If it is detected that the pixel is in the shadow in one of the four images, for that specific pixel, the intensity data from that image will be abandoned, and a modified reflectance map will be used instead of the original map. The image shadow related mapping inaccuracy can then be alleviated.

**Figure 3:**
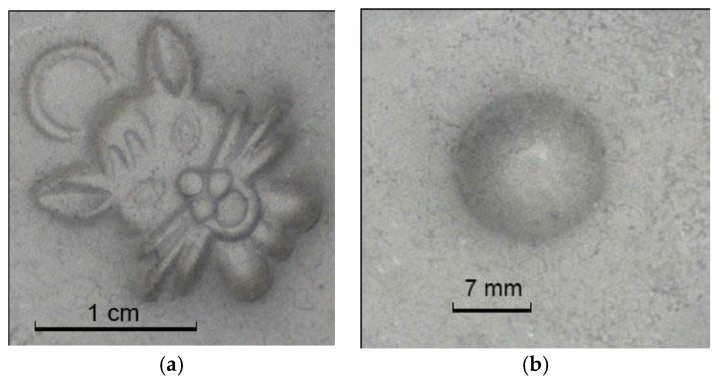
The 2-D surface deformation when the robotic finger touches different objects: (**a**) small mascot; (**b**) rigid sphere.

In the experimental prototype built to validate the proposed method, the image sensor is mounted vertically on top of the sensing layer. The image sensor is equipped with a Tamron lens (AF70-300 mm F/4-5.6 Macro 1:2), which aims at the center of the deformation sensing layer at a distance of 1 m. Four white LEDs in the prototype are evenly spaced on a circle with a radius of 3 cm, and the height difference between the LEDs and the sensing layer is 20 cm. Note that the prototype sensor is built to validate the method proposed, and in a practical sensor implementation in the near future, a macro lens will be used to dramatically scale down the tactile sensor size so it can be fitted into a robotic finger. A CMOS image sensor (CIS) with a dedicated macro lens can provide a minimum object distance of 5 mm, and the circuit board width is expected to be less than 10 mm with a CIS package smaller than 3 mm × 3 mm, which means it can be easily placed in a robotic finger with the size close to the real finger. The typical 2-D deformation images with different objects (a small mascot with a length of about 2 cm, and a rigid sphere with a radius of 7 mm) taken by the prototype sensor are given in Figure 3. The 2-D images are processed using the algorithm in the following section to reconstruct the 3-D surface shape.

## 3. The 3-D Surface Reconstruction Algorithm

Since the thin film on the surface of the sensing layer offers the same BRDF for all objects which the robotic hand grasps, it is reasonable to assume the albedo *ρ* is a constant. The object surface area that needs the 3-D reconstruction can be classified into two types of regions, depending on whether in the shadow. It is assumed in this work that those image regions not in the shadow obey the Lambertian reflectance as the texture depth to identify is much smaller than the distance between the sensing layer and the lens/LEDs, and the miniature LED flashing lights can be viewed as the point lights due to their small sizes [35].

If the object surface vertical depth distribution *z* is described by *z* = *f*(*x*, *y*) in an Euclidean coordinate system, for which (*x*, *y*) is the horizontal coordinate, *z* is the vertical depth, and the image projection is orthographic (the image *x/y* axes coincide with the object *x*/*y* axes), then the local gradient *G*(*x*_0_, *y*_0_) = (*p*(*x*_0_, *y*_0_), *q*(*x*_0_, *y*_0_)) at the location (*x*_0_, *y*_0_) can be expressed as

(2) $$p\left(x_{0},y_{0}\right)={\partial z/\partial x|}_{x0,y0},q\left(x_{0},y_{0}\right)={\partial z/\partial y|}_{x0,y0}$$

The goal of the 3-D surface reconstruction is actually to find the vertical depth *z* for any coordinate on the object surface.

For each coordinate (*x*_0_, *y*_0_), the normal vector $\vec{N}(x_{0},y_{0})$ is defined as (*p*(*x*_0_, *y*_0_), *q*(*x*_0_, *y*_0_), −1)^T^. It would be fair to assume that the surface texture is composed of many small blocks that are approximately flat. In this work, each image block contains only one pixel and its normal vector represents the local slope around that pixel.

The direction of illumination using any of the four LEDs is denoted by a vector *L*, which points from the sensing layer towards the LED. As there are four LED light sources, their directions are described using the three-element vectors *L*_1_, *L*_2_, *L*_3_ and *L*_4_, respectively. For any image taken using the LED with the direction *L_k_* (*k* = 1, 2, 3, 4), *I_k_*(*x*_0_, *y*_0_) denotes the image intensity at the coordinate (*x*_0_, *y*_0_).

### 3.1. Photometric Stereo for Non-Shadowed Regions

According to Horn’s work [36], the image irradiance is a function only dependent on the local surface gradient when the lighting conditions are fixed. So the image intensity at position (*x*_0_, *y*_0_) can be written as *I*(*x*_0_, *y*_0_) = *R*(*G*(*x*_0_, *y*_0_)) = *R*(*p*(*x*_0_, *y*_0_), *q*(*x*_0_, *y*_0_)), where *R* is also called the reflectance map. As there are 4 images for each object, finally we have (*I*_1_, *I*_2_, *I*_3_, *I*_4_) = *R*(*G*) = *R*(*p*, *q*).

**Figure 4:**
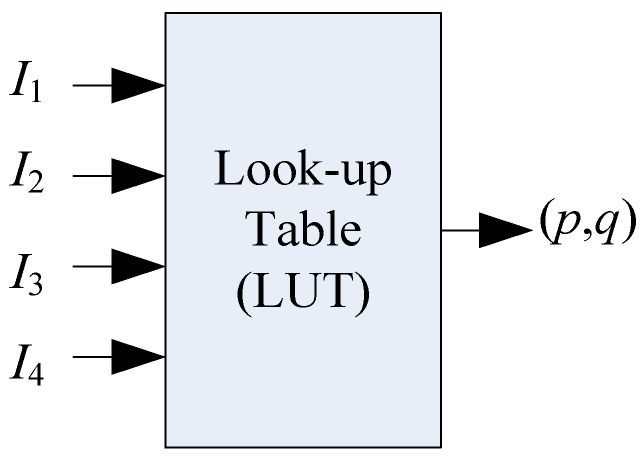
Conceptual diagram of the look-up table.

Similar to Woodham’s method [35], the first step in this work is to use a calibration object with the known shape, size and position (actually known *z* = *f*(*x*, *y*) in this case) to generate the reflectance map *R*. In this work, a rigid sphere with a radius of 7 mm is chosen as the calibration object. In the experiment, the optical system is carefully adjusted to avoid any image shadow in this step. Figure 3b shows the sensing layer film deformation image when the calibration sphere is pressed on it. After this step, a 4-input LUT as shown in Figure 4 is built that relates the gradient *G* and the intensity *I_k_* (*k* = 1, 2, 3, 4), which is actually a portion of the reflectance map *R*. Though the measured *I_k_* using the calibration object cannot fully iterate the *I* space, it does indeed cover most of the images taken by the same tactile sensor. In contrast to the situation using only one LED flashing light in which the *I* space has very limited resolution, the usage of 4 individually controlled LEDs gives a more complicated *I* space with much more resolution steps [29]. The ambiguity of the mapping between *I* and *G* is greatly reduced by using four intensity numbers to find the gradient at any coordinate on the object.

For the object to be identified by the tactile sensor, we can then use the LUT to find the object surface gradient *G* with the measured *I* as the input to the LUT. For any element of the measured *I* of a specific object, we need to find the closest intensity value (actually a four-element vector) in the LUT. This is a nearest-neighbor search problem and the *k-*d tree is used to accelerate the calculation [29]. Since each *I*(*x*, *y*) is a 4-element vector, we need to use a 4-d tree. Then we use the *k*-NN (short for *k* Nearest Neighbor) search algorithm [37] to obtain the gradient corresponding to the nearest reference intensity vector in the LUT, which is denoted as *G*_0_ = (*p*_0_, *q*_0_).

To get a more accurate gradient value, we need to refine the estimated gradient *G*_0_. Firstly, with the assumption of Lambertian reflectance and the fixed illuminating conditions, the reflectance function of the calibration sphere object is modeled using the spherical harmonic basis functions [38]

(3) $$R(\vec{N})=\sum_{n=0}^{\infty }\sum_{m=-n}^{n}l_{n,m}Y_{n,m}(\vec{N})$$

in which *Y_n,m_* is the spherical harmonic and *l_n,m_* is the spherical harmonic coefficient of the incident illumination. As *l_n,m_* is a constant which decays rapidly for *n*, about 99 percent of the energy is contained within *n* ≤ 2 [39]. Thus, Equation (3) can be reduced to Equation (4), in which only 9 coefficients are estimated using the least squares method.

(4) $$R(\vec{N})=\sum_{n=0\;}^{2}\sum_{m=-n}^{n}l_{n,m}Y_{n,m}(\vec{N})$$

Secondly, the intensity in the neighborhood of (*p*_0_, *q*_0_) is approximated using a first-order Taylor series expansion,

(5) $$R(p,q)=R(p_{0},q_{0})+J(p,q)(\begin{array}{l} p-p_{0}\\ q-q_{0} \end{array})\;$$

in which *J* is the matrix of the first partial derivatives of Equation (4) at the coarsely estimated gradient *G*_0_ = (*p*_0_, *q*_0_), *R*(*p*_0_, *q*_0_) is calculated by the spherical harmonic basis function, and *R*(*p*, *q*) is the accurate intensity of the gradient mapping. A more accurate gradient *G* = (*p*, *q*) can be then obtained by inverting the Taylor series expansion as Equation (6). As *J* is not an invertible matrix, a pseudo inverse matrix *J*^+^ can be used instead.

(6) $$\left(\begin{array}{l} p\\ q \end{array}\right)=J^{+}\left(R(p,q)-R(p_{0},q_{0})\right)+\left(\begin{array}{l} p_{0}\\ q_{0} \end{array}\right)$$

### 3.2. Detection of Shadow

As analyzed previously, the image shadow is of great concern for the 3-D surface reconstruction. The shadow is almost inevitable when the object surface fluctuation is large and steep. The algorithm in Section 3.1 can only be used to deal with those image blocks not in the shadow. In the rest of this subsection, it is explained how to handle the image blocks in the shadowed regions.

A widely used method to detect the shadow is to compare a pixel’s intensity to a threshold. It can be shown that the non-shadowed blocks behave with the Lambertian reflectance [35], while those in the shadowed regions are non-Lambertian. Therefore, the shadow can be treated as the disturbance in the Lambertian photometric function. Again, the assumption of the point lights is still valid in the following discussion.

Firstly, consider an image block with a unit normal vector $\vec{n}=\vec{N}/\left\|\vec{N}\right\|$ which is illuminated by four light sources *L*_1_, *L*_2_, *L*_3_ and *L*_4_. For the non-shadowed blocks that behave with the Lambertian reflectance, the intensities can be expressed as [35],

(7) $$I_{k}=\rho L_{k}\vec{n}\;k=1,2,3,4$$

in which $\vec{n}=\frac{1}{\sqrt{p^{2}+q^{2}+1}}\left(p,q,-1\right)$.

In a 3-D space, any four vectors are linearly dependent. Therefore, the four direction vectors of the light sources are also linearly dependent, which means that there exist the coefficients *a_k_*, *k* = 1, 2, 3, 4 to satisfy the following equation

(8) $$a_{1}L_{1}+a_{2}L_{2}+a_{3}L_{3}+a_{4}L_{4}=0$$

Then by multiplying both sides of Equation (8) with the constant albedo *ρ* and the unit normal $\vec{n}$, we can have

(9) $$\rho a_{1}L_{1}\vec{n}+\rho a_{2}L_{2}\vec{n}+\rho a_{3}L_{3}\vec{n}+\rho a_{4}L_{4}\vec{n}=0$$

By combing Equation (7), Equation (9) is simplified to Equation (10) which demonstrates that the linear dependence of light source directions leads to the linear dependence of the intensities.

(10) $$a_{1}I_{1}+a_{2}I_{2}+a_{3}I_{3}+a_{4}I_{4}=0$$

Equation (10) can also be written in the vector form as Equation (11), which is just the Lambertian photometric function.

(11) a⋅I=0

The vector ***a***= (*a*_1_, *a*_2_, *a*_3_, *a*_4_) can be calculated from the illumination matrix I*I^T^ as an eigenvector which corresponds to the zero eigenvalue [40]. This is a criterion to determine whether the block is in the shadow or not. As the image data obtained contains noise and the coefficients ***a*** calculated may have slight deviations, the result of Equation (11) cannot be exactly equal to zero even for a non-shadowed block. Therefore, a threshold *λ* should be chosen for the shadow detection. A block is considered to be in the shadow if the dot product given by Equation (11) is larger than the threshold *λ*.

### 3.3. Gradients of Shaded Blocks

With the processing in Section 3.2, the image blocks (actually only one pixel in each block) are classified into two types to tell whether they are in the shadow. For those not in the shadow, the algorithm presented in Section 3.1 is available to calculate the local gradient. In this subsection, it will be illustrated how to obtain the gradients of the blocks in the shadow.

In this work it is assumed that for any coordinate on the object surface, only one element of the four measured intensity numbers is affected by the shadow, and this is true for almost all the objects with modest surface gradients in the experiment. Then we can use the other three intensity numbers to find the gradient for this specific coordinate. As the intensity number affected by the shadow is small, we can choose the three largest ones out of all the four intensity numbers for one specific block to carry on the calculation. Because we abandon the darkest element of the four-element intensity vector, the LUT created in Section 3.1 cannot be used. We need to reconstruct a table for the three-element intensity case. For example, if the fourth element of the intensity vector is the smallest, a new LUT will just use the other three elements as the input to the LUT. Thus, we need to re-build four new LUT’s with the three-element intensity. The data structured *k*-d tree is then applied to the new LUT’s and in this case *k* equals to three as the input. Finally, the *k*-NN search is used to find the three-element reference intensity vector nearest to the element-reduced intensity input to find the corresponding gradient value.

### 3.4. Depth from Gradients

The goal of the 3-D reconstruction is to find the vertical depth *z* for any coordinate on the object surface. With the gradient calculation for both the non-shadowed and shadowed image blocks, we have the gradient field *G*(*x*, *y*) = (*p*(*x*, *y*), *q*(*x*, *y*)). The last step is to obtain the depth information *z*(*x*, *y*) from *G*(*x*, *y*). We define the error function *E*(*z*; *p*, *q*) expressed as

(12) $$E(z;p,q)={\left(\frac{\partial z}{\partial x}-p\right)}^{2}+{\left(\frac{\partial z}{\partial y}-q\right)}^{2}$$

It is noted that the error function *E* is a function based on the coordinate (*x*, *y*). Consequently, the problem of finding *z* (*x*, *y*) can be viewed as the minimization of the following cost function

(13) Cost=min∬E(z;p,q)dxdy 

In this work, the Frankot–Chellappa algorithm [41] is used and the depth matrix can be calculated as

(14) $$Z=F^{-1}\left\{-j\frac{\frac{2\pi u}{N}F\left\{p\right\}+\frac{2\pi v}{M}F\left\{q\right\}}{{\left(\frac{2\pi u}{N}\right)}^{2}+{\left(\frac{2\pi v}{M}\right)}^{2}}\right\}$$

in which *F*{} and *F*^−1^{} represent the discrete Fourier Transform (DFT) and the inversed DFT (IDFT) operations, *M* and *N* are the image length and width, and *u* and *v* are the 2-D index with range of [−*N*/2, *N*/2] and [−*M*/2, *M*/2], respectively.

### 3.5. Prcoessing Flow

**Figure 5:**
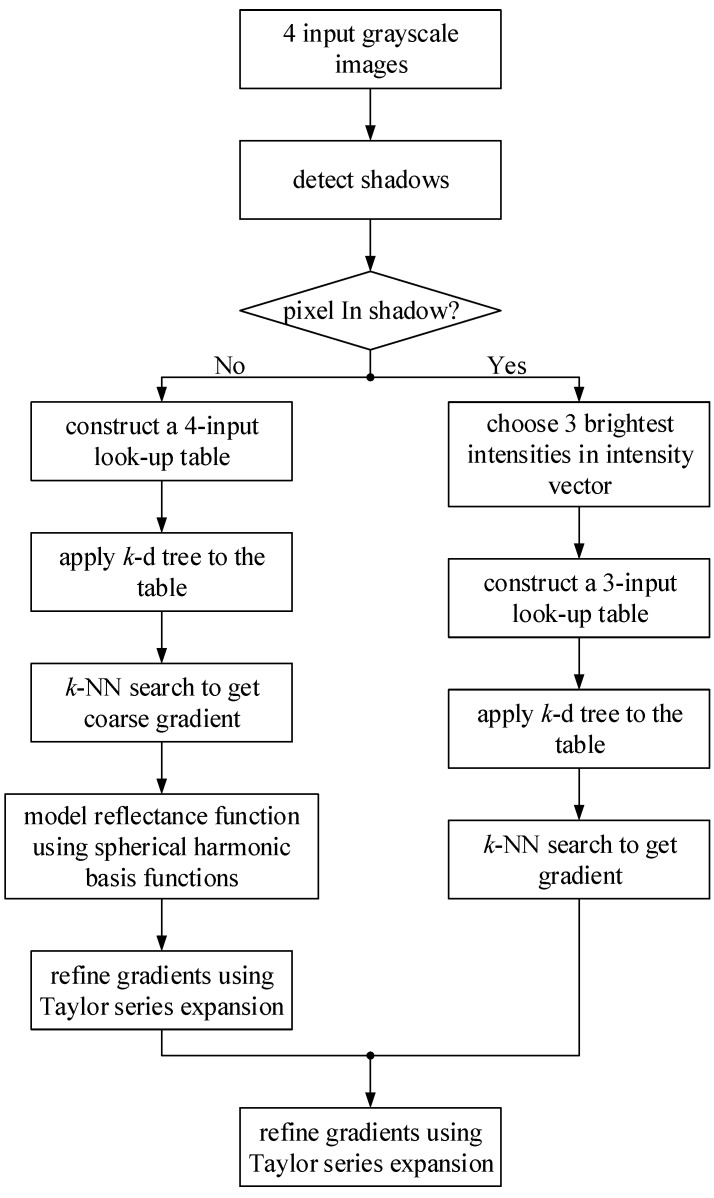
Flow chart of the entire 3-D reconstruction algorithm.

The entire processing flow of the 3-D reconstruction algorithm is illustrated in Figure 5. For each pixel coordinate, the first step is to determine whether the pixel is in the shadow.

(a) If it is a non-shadowed pixel, it complies with the Lambertian reflectance and we can use the photometric stereo method to find the local gradient. A LUT is created that relates the image intensity measured from the 4 images taken using different flashing LEDs and the gradient based a known object. Then the *k*-NN search algorithm is used to find the gradient value with the measured intensity. In order to accelerate the searching speed, the data structured *k*-d tree is applied to the LUT. A coarse gradient value is obtained with the above steps, and the next step is to refine it. As the Lambertian object can be approximated by a low-dimensional linear subspace, the reflectance function of the calibration object is modeled using the spherical harmonic basis functions. The accurate gradient values are obtained by a first-order Taylor series expansion in the neighborhood of the coarse gradient values.
(b) If any pixel is identified as shadowed, a different flow is used for the gradient calculation. By assuming that there is only one element of the four-element intensity vector affected by the shadow and its intensity is the lowest, we can choose the three largest elements out of the four intensity numbers to reconstruct the local gradient, which effectively avoid the influence of shadow. As we abandon the darkest intensity, a new LUT is used which does not contain the same dimension as that of the darkest intensity. Again, we make use of the *k*-NN search algorithm to get the gradient value. For the pixels in shadow, there is no gradient refining processing after the *k*-NN search. Actually, for the pixels in shadow, the intensity data dimension is reduced to 3 in contrast to the 4-dimension intensity data for pixels not in shadow. It is not surprising that the gradient refining benefit weakens with the reduced data dimension/redundancy. To reduce the algorithm complexity, the gradient refining process is not performed for the pixels in the shadow.

The last step of the algorithm is to calculate the depth matrix for the specific object from the gradient matrix using the Frankot–Chellappa algorithm [41].

## 4. Implementation Results

To verify the performance of the proposed 3-D reconstruction method, both the computer simulation and the experiment on the prototype tactile sensor have been carried out.

### 4.1. Simulation Results

For the purpose of simulation, 3D Studio Max software is used to build a model to emulate the tactile sensor, including the thin film deformation, the flashing lights and the image sensor. According the proposed algorithm, a set of LUT’s (one with four-element intensity vectors and the other four with three-element intensity vectors) which relate the object gradient and the measured intensity data, are built based on four non-shadowed images of a known rigid sphere as the calibration object. Then the 3-D reconstruction is tested on a set of objects, including a “unknown” rigid sphere, a teapot and a ceramic cat.

**Figure 6:**
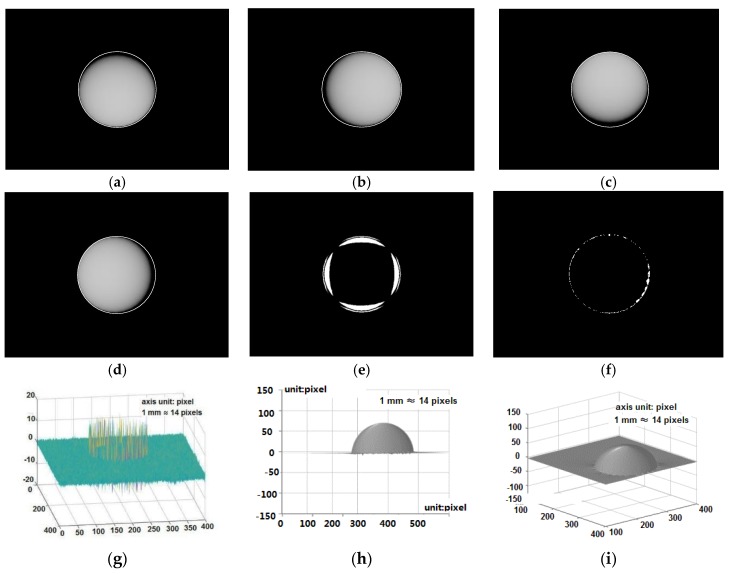
Simulated 3-D reconstruction of an “unknown” rigid sphere: (**a**–**d**) four sphere images with shadows, (**e**) shadow detection, (**f**) 3-D reconstruction error (absolute value projected to the bottom plane), (**g**) 3-D reconstruction error (absolute error <15 pixels, in contrast to the sphere height of 70 pixels), (**h**,**i**) 3-D reconstructed sphere.

The 3-D reconstruction of the “unknown” sphere with a radius of 5 mm is shown in Figure 6. Figure 6a–d give the sphere images with the shadow. The shadow detection result is given in Figure 6e. The absolute value of the 3-D reconstruction error projected to the bottom plane is given in Figure 6f with the white regions representing the reconstruction error larger than 10%. As show in Figure 6g, the reconstruction error peaks at the edge of the sphere, and the maximum reconstruction error is less than ±15 pixels, in contrast to the sphere height of 70 pixels which is actually 5 mm. The 3-D reconstruction results of this object are shown in Figure 6h,i. Through this simulation, it is found that the proposed tactile sensor may still introduce error for object surface regions with very steep gradient change, but much smaller than that without the proposed method.

The simulated reconstruction error using the proposed shadow detection method is compared to that without the shadow detection in Table 1. The maximum reconstruction error is ±15 pixels with the proposed shadow detection, while the maximum error reaches about ±23 pixels without the shadow detection. The number of pixels with >10% reconstruction error is reduced to 1.62% of the number of pixels covered by the sphere, in contrast to 5.73% without the shadow detection.

**Figure 7:**
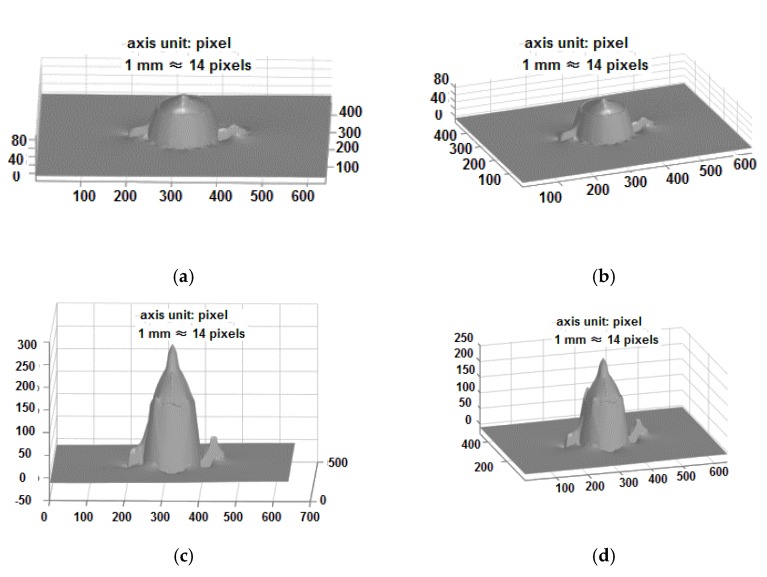
The 3-D reconstruction of a simulated teapot: (**a**,**b**) with the proposed shadow detection, (**c**,**d**) without shadow detection.

Figure 7 compares the 3-D reconstruction of a simulated teapot with the proposed shadow detection (Figure 7a,b) to that without the shadow detection (Figure 7c,d). It is clearly shown that the shadows can greatly affect the reconstruction of the object steep edges, and the reconstruction error will be large if the shadow detection is not applied.

**Figure 8:**
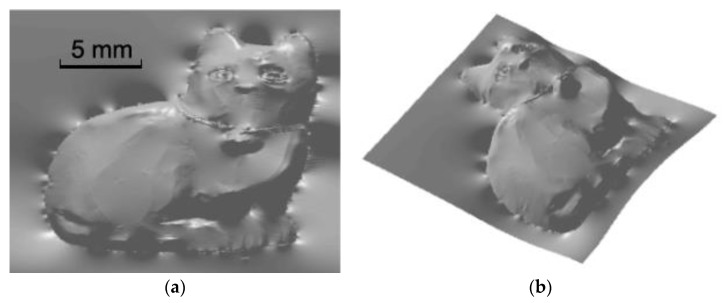
Reconstructed cat using the proposed method (two view angels): (**a**) top view, (**b**) rotated view.

The 3-D reconstruction of a simulated ceramic cat using the proposed method is shown in Figure 8. And this simulation again validates the effectiveness of the proposed method.

### 4.2. Experimental Results

**Figure 9:**
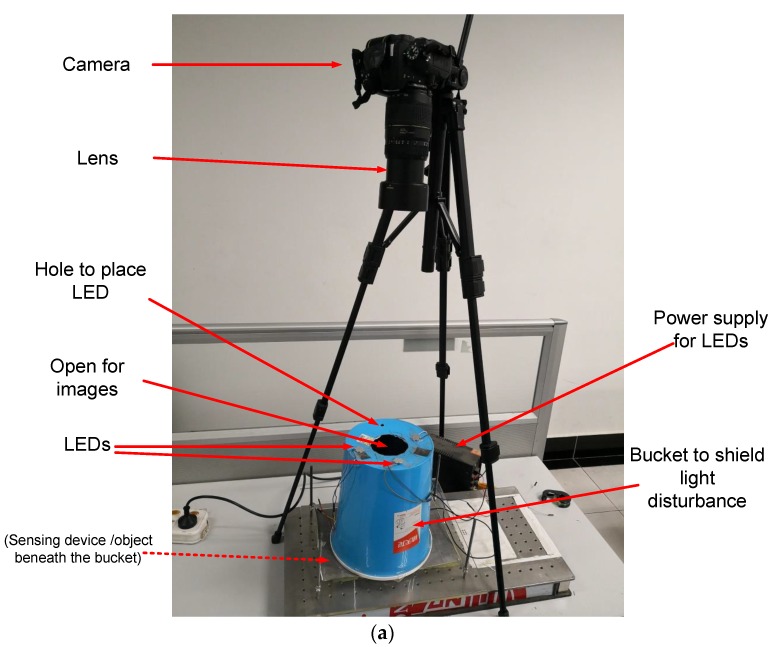

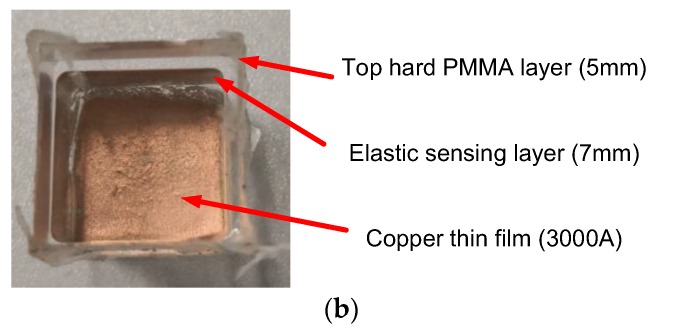
Experiment setup: (**a**) image acquisition, (**b**) sensing device with sensing film at the bottom.

The experiments have been conducted using the tactile sensor prototype to further validate the proposed method. The experiment setup is shown in Figure 9a. The blue plastic bucket with the bottom up is used to shield the sensing device and the object from the environment light disturbance. The 4 flashing LEDs are placed on the bottom of the bucket to illuminate the sensing device and the object beneath the bucket, and the LEDs are 20 cm above the sensing device. The object images are taken using a 4928 × 3264 Nikon D7000 camera mounted on a tripod, and the camera is vertically on top of an open at the center of the bucket bottom. The camera is equipped with a Tamron lens (AF 70–300 mm). The images are cut to 640 × 480 pixels to remove the irrelevant regions. The sensing device composed of a 5-mm PMMA layer, a 7-mm elastic sensing layer and a 3000-A copper film is shown in Figure 9b, and its size is about 4 cm × 4 cm.

**Figure 10:**
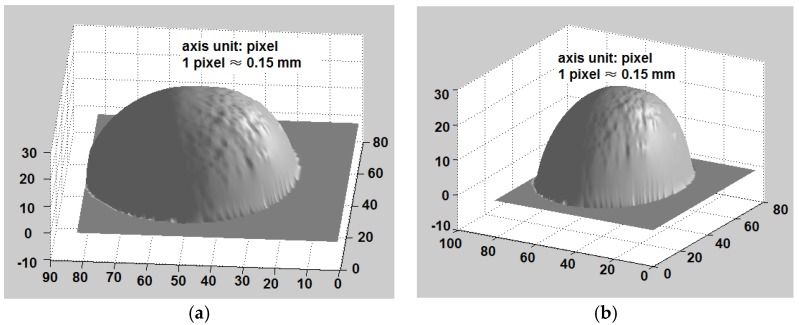
The 3-D reconstruction of a rigid sphere (two different view angles): (**a**) from the upper top direction, (**b**) from the right upper top direction.

**Figure 11:**
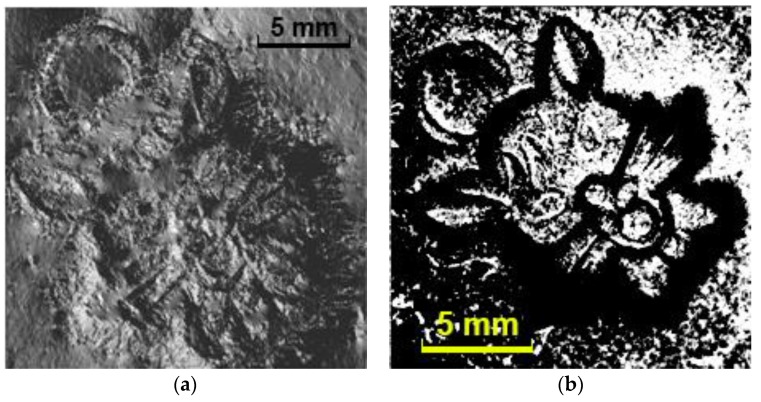
The 3-D reconstruction of a 2-cm mascot: (**a**) reconstructed image, (**b**) shadow detected using the proposed shadow detection method (the white areas/dots represent the shadow).

Figure 10 shows the 3-D reconstruction results of a rigid sphere with a radius of 5 mm (not the calibration one for the LUT generation). It is seen that the rigid sphere can be correctly reconstructed. However, the reconstruction error of a 2-cm mascot (the same object shown in Figure 3a) is more than expected, as shown in Figure 11. Actually, the reconstruction error is quite large in the image regions with too many steep stages. A further study reveals that the possible cause is that the sensing layer is not soft enough and it cannot perceive the small details of the complicated object surface.

In general, the experiments have validated the proposed 3-D reconstruction method for objects with not complicated surface structure. Compared to the methods without the shadow processing, the proposed method has some extra requirements. Firstly, the proposed tactile sensor needs to take four images for each object to sense. A high speed camera is required to maintain a certain sensing speed, and the camera frame rate needs to be 4 times the refresh rate of the tactile sensing. The maximum refresh rate of this tactile sensor is only 7.5 Hz using a CMOS image sensor with a frame rate of 30 frame/second. Secondly, the computation complexity is increased with the shadow detection procedure. Thirdly, in addition to the four-input LUT, 4 three-input LUT’s are required to complete the inverse reflectance mapping, which means extra memory space. To sum up, the proposed method solves the shadow problem at the cost of hardware and computation overhead.

## 5. Conclusions and Future Work

In this paper, an optical tactile sensor with 3-D surface reconstruction is proposed for robotic fingers. The presented method takes multiple images of the object with four individually controlled flashing LEDs that provide different illuminating directions. In contrast to the GelSight sensors in the literature, the key difference of this work is to check if any pixel is affected by the illumination shadow first and to abandon the intensity data disturbed by the shadow caused by one of the four LEDs. The shadow related inaccuracy is alleviated using this method, which is especially important for the object surface with the large and steep fluctuation. Both the simulation and experimental results have successfully validated the proposed 3-D surface reconstruction method.

The authors are continuing working on this tactile sensor to improve the 3-D reconstruction performance. Actually, due to the reason that the softness of the sensing layer is not as good as expected, the prototype tactile sensor can only sense the objects with simple surface texture in the experiment. In the future, the physical property of the deformation sensing layer will be improved such that the proposed tactile sensor can also work for objects with very complicated surface structure. Also, efforts will be made to scale down the tactile sensor size by using a dedicated macro lens with a minimum object distance of about 5 mm, and a CMOS image sensor with the package size of less than 3 mm × 3 mm. The ultimate goal of this work is to fit the tactile sensor in the real small robotic fingers.

## Figures and Tables

**Table 1:** Reconstruction error comparison.

Method	Max Error ^1^	Pixels Percentage with >10% Error ^2^
w/shadow detection	±15 pixels	1.62%
w/o shadow detection	±23 pixels	5.73%

^1^ Sphere height: 70 pixels; ^2^ Number of pixels with >10% error divided by the sphere area.
